# Land-Applied Goat Manure as a Source of Human Q-Fever in the Netherlands, 2006–2010

**DOI:** 10.1371/journal.pone.0096607

**Published:** 2014-05-02

**Authors:** Tia Hermans, Leonne Jeurissen, Volker Hackert, Christian Hoebe

**Affiliations:** 1 Alterra, Wageningen UR, Wageningen, The Netherlands; 2 Department of Sexual Health, Infectious Diseases, and Environmental Health, South Limburg Public Health Service, Geleen, The Netherlands; 3 Department of Medical Microbiology, School of Public Health and Primary Care, Maastricht University Medical Center, Maastricht, The Netherlands; INIAV, I.P.- National Institute of Agriculture and Veterinary Research, Portugal

## Abstract

Studies have shown a link between Q-fever positive farms (QFPFs) and community cases of human Q-fever. Our study is the first to investigate the potential role of contaminated land-applied manure in human Q-fever, based on a large set of nationwide notification and farm management data. Time between manure application and disease onset in geographically linked notified human cases coincided with the incubation period of Q-fever. Proximity of contaminated land parcels predicted human cases better than proximity of QFPFs (80% vs. 58%, 0–5 km in 2009). Incidence around QFPFs and contaminated land parcels decreased with distance, but not around non-contaminated land parcels. Incidence was higher around contaminated land parcels than non-contaminated land parcels (RR = [10],95%CI = [7], [1]–[14,2]). Our findings deliver evidence that, apart from QFPFs, land-applied contaminated manure may be another source of human Q-fever.

## Introduction

In 1975, Q-fever in humans was made a notifiable disease in the Netherlands [1]. Between 1975 and 2006 the annual number of human cases varied between 0 and 32 per year countrywide [2]. From 2007 to 2010, the Netherlands faced large seasonal outbreaks of human Q-fever (more than 4000 notifications) with the highest peak in 2009 [3]. From its start, experts identified dairy goat farms with Q-fever induced abortion waves as the primary source. By the end of 2012, Dutch authorities declared the Q-fever outbreak over after about 50,000 pregnant goats had been culled and the remaining dairy goats had been given mandatory vaccination.

Q-fever is a zoonosis caused by the bacterium *Coxiella burnetii.* Except for New Zealand, *C. burnetii* has a worldwide distribution in domestic and wild animals, but its transmission to humans is mostly associated with sheep and goats [4]. During parturition in sheep and goats, birth products of infected animals (and possibly other infected animal excretions), containing billions of bacteria [5], mix with the deep-litter manure in the stables. Contaminated manure may subsequently dry up and aerosolize, followed by wind-borne transmission [6], [7], [8]. In humans, Q-fever is essentially an airborne infection resulting from the inhalation of contaminated aerosols [9], [10], [11], [12]. Exposure to a highly aerosolized dose of C. burnetii is currently assumed to be the most important risk factor for human infection [13].

In the Netherlands, the main source of aerosolized bacteria has been attributed to Q-fever positive dairy-goat farms [14], [15]. Several case studies have shown a spatial relationship between residential locations of human cases and infected small ruminant farms located nearby [14], [15], [16], [17]. However, a nationwide analysis on the spatial relationship between human cases and Q-fever positive farms housing dairy goats or sheep with clinical symptoms found clusters of human cases for only 14 out of 29 separately investigated farms [18]. Moreover, more than 40% of the Q-fever patients could not be related to a Q-fever positive farm within a 5-km distance. Further study was done to assess how environmental conditions around Q-fever infected farms might add to or reduce the transmission of Q-fever [19]. It was found that low vegetation density and dry soil around a farm increase the probability of transmitting Q-fever to humans.

Dutch dairy goats are housed in so-called ‘deep-litter stables’, i.e., stables where farmers top up existing manure with fresh hay and straw every few days. After several months, the manure layer becomes so large that is has to be removed. Land-owning farmers apply the deep-litter manure to their land-. Farmers without land, or those without sufficient land, transport all or part of the manure to other – mostly arable – farms which are often located outside the region. Deep-litter manure is mostly applied with a manure spreader on bare soils or on low vegetation before the growing season and according to Dutch manure legislation [20]. Several studies have shown that manure from Q-fever positive dairy goat farms may contain high concentrations of *C. burnetii* [5], [21], [22], suggesting that land-applied manure from such farms may be an important additional source of human Q-fever, at least if the manure is applied during or shortly after the lambing season due to large numbers of bacteria in birth products that mix with the deep-litter manure [5]. A recent epidemiological study has provided firm evidence linking manure application and livestock operations to human disease [23].

The objective of our study is to assess the potential role of dairy-goat manure from Q-fever positive farms land-applied during or shortly after the lambing season as a source of human Q-fever during the large outbreak in the Netherlands in the period 2006–2010.

## Materials and Methods

### Study Design

To substantiate our hypothesis, we used a comprehensive set of national epidemiological data, including all manure transports that took place in the Netherlands from farms with Q-fever in the study period, all lambing periods registered on farms with Q-fever in the study period, and individual onset of illness dates for all notified human cases in the study period, to establish 1) the temporal sequence of events regarding lambings, manure transports, and human cases; 2) the percentage of human cases that could be related to at least one Q-fever positive farm (QFPF) or contaminated land parcel at three distance classes; and 3) human incidence at three distance classes around QFPFs without land, around QFPFs with land, around contaminated land parcels and around non-contaminated parcels.

### Data Collection

#### Goat farms

For this study, we used the Geographic Information Agrarian Farms (GIAB) database [24] to extract data on the location of dairy goat and sheep farms from 2006 till 2010. Data on goat and sheep farms with clinical Q-fever (abortion waves with more than 5% abortion rate in pregnant animals, subsequently confirmed by polymerase chain reaction (PCR) testing in goats) for the period 2006–September 2009 were provided by the National Animal Health Service (GD). From October 2009 onwards, Bulk Tank Milk (BTM) monitoring upon dairy goat farms was mandated. Data on farms that tested positive in BTM monitoring were available from the Dutch Food and Consumer Product Safety Authority (nVWA) and from the Central Veterinary Institute (CVI). Data from GD, nVWA and CVI were combined into one dataset. This dataset included the method used for the diagnosis of Q-fever (clinical or BTM) and the date of notification, i.e., the date on which the farm tested positive for the first time. Once a farm had tested positive (clinical or BTM), it was considered a QFPF. Around the turn of 2009/10, mandatory culling of all pregnant goats on QFPFs took place [2]. Since parturition from infected goats may be considered the primary source of contamination of deep – litter manure with *C. burnetii*, a QFPF was no longer included in our analysis once culling of pregnant goats had taken place on that particular farm. The combined dataset yielded 117 unique QFPFs, 113 of which were goat farms and four of which were sheep farms. 29 Farms were clinically affected, 16 of which were also BTM-positive, while 88 farms were BTM-positive only.

Data on the number of lambs born per month on farms with Q-fever between 2006 and 2010 was provided by the Dutch National Service for the Implementation of Regulations (DR), a semi-independent governmental organization responsible for the implementation of numerous European and Dutch regulations. For one farm, data were limited to the number of lambs born per annum, and January was assigned as that farm’s lambing period.

Data on manure transport from farms with Q-fever between 2006 and 2010 were provided by DR. From these data, we extracted the location of manure production, the date of transport and the location of manure disposal. Registration of farm-to-farm manure transport, irrespective of distance between farms, was - and still is - mandatory in the Netherlands, also during the study period (DR). The date of transport was assumed to be the date of application. Locations of manure disposal were available as *x-,y*-coordinates based on GPS (86%) and on 6-digit postal code (6PC) centroid (14%) of the receiving address. No dates of manure disposal were available for farmland owned by the farmer because those farms applied their goat manure to their own land parcels and had no legal obligation to register the date and land parcel of disposal. For 2009, land parcels were identified with the Dutch land parcel registration (BRP) database [25].

#### Human cases of Q-fever

Acute human Q-fever is a notifiable disease in the Netherlands. To allow geo-referencing of human cases, the regional Public Health Services provided anonymised data on 3958 Q-fever patients notified between January 1, 2007 and August 1, 2010. Data were limited to the date of illness onset and the postal code of each patient’s residential address. Ten patients (0.003%) for whom no postal code was available and 141 patients (0.04%) for whom no onset date was known were excluded from the analysis. For 3754 patients (95%), high-resolution 6PC were available, for 28 patients 5-digit postal codes (5PC), and for 25 patients 4-digit postal codes (4PC). For all 3807 patients, the centroid of each corresponding 6PC, 5PC, or 4PC area was used for analysis.

#### Population data

Dutch population data were obtained from CBS-Statline [26]. The Netherlands had 16.5 million residents at the time of the study. The number of residents per 4PC area as of January 1, 2009 was used for analysis. Residents were spatially attributed to their 4PC centroid. 4PC areas were defined according to the Bridgis 2008 geometry. The Netherlands has 4026 4PC areas, covering a median surface of 5.4 km^2^ (range 0.1–137 km^2^) each.

### Data Analysis

#### National temporal assessment

Monthly numbers of manure transports from farms with Q-fever were aggregated on a national level from 2006 till 2010 and correlated with monthly numbers of lambs born and monthly numbers of human Q-fever cases. Multiple transports from the same farm on the same day to the same location were counted as one trip. The number of human cases per month was calculated as the total number of monthly notifications, based on the date of illness onset.

#### Temporo-spatial association between human cases and QFPFs or contaminated manure destinations

Manure transports preceding the date when a farm was labelled positive were excluded from the temporo-spatial analysis. Manure application locations were classified by their *C. burnetii* contamination status, based on the following assumption: manure from a QFPF spread during the lambing period or within three months after lambing was considered contaminated, yielding a contaminated land parcel (i.e. 59% of all transports) while all other manure transports were considered non-contaminated. This assumption is in line with the analyses of *C. burnetii* survival in dung hills on two QFPFs [21]. For land parcels owned by farmers with a QFPF and thus without a known manure transport date, the land parcels were considered contaminated during the time the farm was considered infectious.

Human cases were geographically linked to a QFPF or contaminated land parcel based on a temporal and a spatial criterion. Human cases were only considered to be exposed to a QFPF or contaminated land parcel if the time between QFPF or contaminated land parcel designation and subsequent patient illness onset of Q-fever had not exceeded 180 days. The 180-day period was based on findings from a single-point source outbreak where the vast majority of patients (85%) appeared within six months following lambing on the QFPF [15]. Cases based on this criterion were classified according to their distance to the farm or land parcel in question (within 2.5, 5, or 10 km). A relationship was considered unlikely for distances exceeding 10 km [18]. We calculated the percentages of human cases that could be geographically linked to at least one contaminated land parcel or one QFPF, or both, and compared annual percentages between these three groups. QFPF are divided into farms with and without land.

#### Human incidence near QFPFs and contaminated land parcels

We used GIS (ESRI, ArcGIS 10.0) to determine the number of human cases meeting the 180-day criterion and the number of residents within three distance zones surrounding each QFPF, each contaminated land parcel and each non-contaminated land parcel: 0–2.5 km, 2.5–5 km, and 5–10 km. Incidence was calculated as the number of notified human cases per 100,000 residents. Relative risks (RR) were calculated as the incidence around QFPFs divided by the incidence around contaminated land parcels, and as the incidence around contaminated land parcels divided by the incidence around non-contaminated land parcels. As relative vicinity of contaminated land-owned parcels to QFPFs might introduce bias, we performed a sensitivity analysis where we recalculated incidences and corresponding relative risks excluding all land-owned parcels. The analyses of human incidence were limited to 2009, because 2009 provided the most comprehensive data on patients (including hospitalization) and on contaminated farms.

## Results

### Sequence of Lambing, Manure Transports and Human Cases

**Figure 1:**
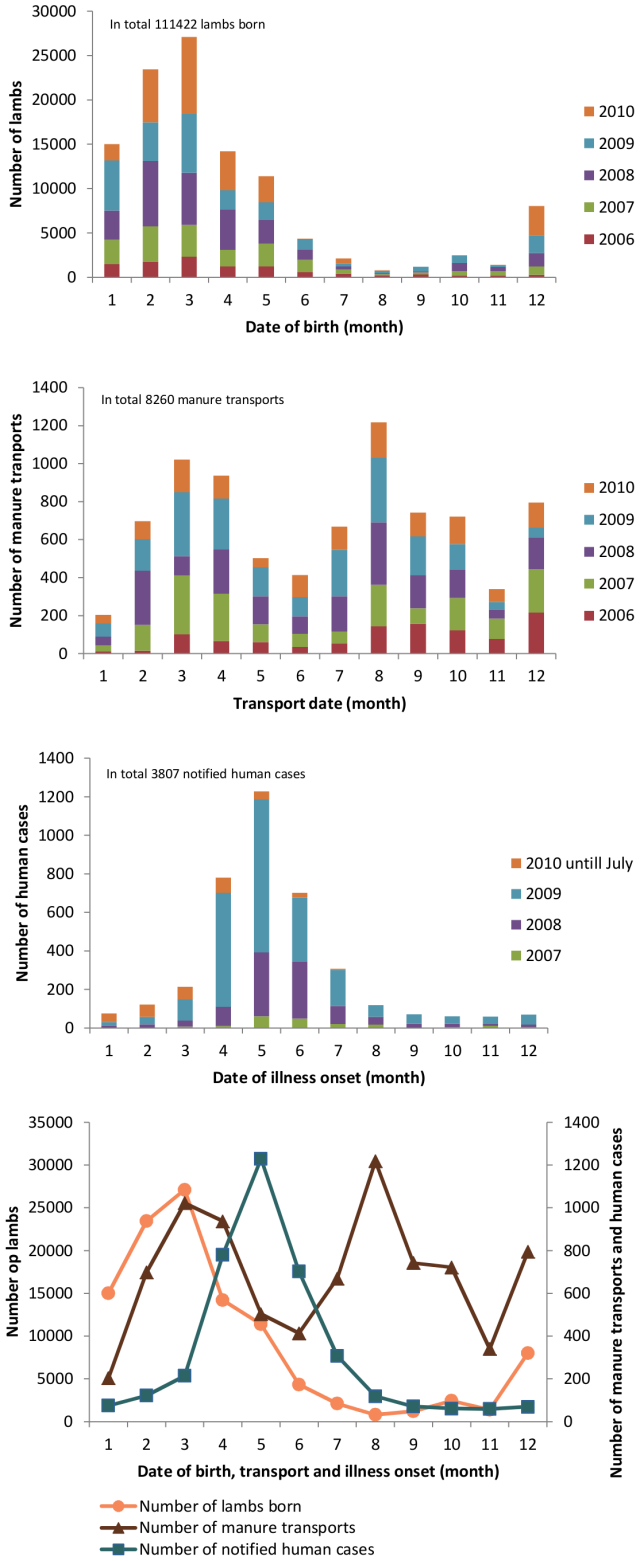
Timeline of lambing and manure application of farms with Q-fever irrespective the year of detection in the Netherlands, 2006–2010, and human Q-fever cases, 2007–August 2010.

Figure 1 shows the timeline of events over a one-year period, aggregated over the study period: lambing peaked in February/March, a period accounting for 45% of all lambings, and declined sharply in April while manure application peaked in March/April, a period accounting for 52% of manure transports up until June; human cases peaked in May. Manure applied in late summer caused a second peak in August.

### Temporo-spatial Association between Human Cases and QFPFs or Contaminated Manure Destinations

Table 1 shows annual numbers and percentages of notified human cases related to at least one QFPF, one contaminated land parcel or one non-contaminated land parcel within distances of 2.5 km, 5 km, or 10 km. The number and percentage of human cases associated with a nearby contaminated land parcel was consistently higher than that associated with a QFPF, regardless of the year or the distance class.

**Table 1:** Annual numbers and percentages of notified human cases over three cumulative distance classes, associated with at least one QFPF, one contaminated land parcel or one non-contaminated land parcel.

		Human cases associated with					
Distance class (km)	Human cases	QFPFs		Contaminated landparcels		Non-contaminatedland parcels	
	No	No	*%*	No	*%*	No	*%*
**0–2.5**							
2007	192	55	*29*	66	*34*	0	*0*
2008	980	113	*12*	316	*32*	26	*3*
2009	2309	618	*27*	1174	*51*	32	*1*
2010	325	83	*26*	107	*33*	20	*6*
**0–5**							
2007	192	69	*36*	103	*54*	10	*5*
2008	980	310	*32*	598	*61*	82	*8*
2009	2309	1331	*58*	1844	*80*	195	*8*
2010	325	162	*50*	200	*62*	54	*17*
**0–10**							
2007	192	147	*77*	167	*87*	86	*45*
2008	980	764	*78*	914	*93*	294	*30*
2009	2309	2157	*93*	2253	*98*	655	*28*
2010	325	269	*83*	295	*91*	144	*44*

Distinguishing between QFPFs with land and those without land, the number and percentage of human cases associated with a QFPF with land was consistently higher than that associated with farms without land, regardless of the year or the distance class (Table 2). Only 1.1% (25/2309) of human cases that were notified countrywide in 2009 were not related to a QFPF or contaminated parcel within a distance of 10 km. An overview of the number of QFPFs, QFPFs with and without land, contaminated and non-contaminated land parcels, potentially associated with the number of human cases, based on the temporal criterion of 180 days only for the different years, is given in Table 3.

**Table 2:** Annual numbers and percentages of human cases over three cumulative distance classes, associated with at least one QFPF with land, one QFPF without land or both within three distance classes.

		Human cases associated with					
Distance class (km)	Human cases	QFPF without land		QFPF with land		Both	
		No	*%*	No	*%*	No	*%*
**0–2.5**							
2007	192	1	*1*	54	*28*	0	*0*
2008	980	5	*1*	108	*11*	0	*0*
2009	2309	94	*4*	502	*22*	22	*1*
2010	325	20	*6*	59	*18*	4	*1*
**0–5**							
2007	192	4	*2*	65	*34*	0	*0*
2008	980	64	*7*	246	*25*	0	*0*
2009	2309	231	*10*	851	*37*	249	*11*
2010	325	35	*11*	100	*31*	27	*8*
**0–10**							
2007	192	5	*3*	122	*64*	20	*10*
2008	980	63	*6*	499	*51*	202	*21*
2009	2309	111	*5*	858	*37*	1188	*51*
2010	325	19	*6*	118	*36*	132	*41*

**Table 3:** Annual numbers of QFPFs, QFPF with and without land, contaminated and non-contaminated land parcels, potentially associated with the number of human cases in different years, based on the temporal criterion*.

Year	Humancases	QFPFs	QFPF withoutland	QFPF with land	Contaminatedland parcels	Non-contaminatedland parcels
	No	No	No	No	No	No
2007	192	16	5	11	350	77
2008	980	24	6	18	628	183
2009	2309	107	25	82	2464	505
2010	325	107	25	82	1593	1158

*Temporal criterion = 180 days.

### Human Incidence around QFPFs, Contaminated Land Parcels and Non-contaminated Land Parcels

Table 4 shows human incidences in 2009 in three distance zones surrounding QFPFs, contaminated land parcels, and non-contaminated land parcels. Incidence was significantly higher around QFPFs than around contaminated land parcels in all distance zones (RR = 2.7, 95%CI = [2,5–3,0]). Incidence around contaminated land parcels, in turn, was significantly higher than around non-contaminated land parcels. At least in the 0–2.5-km and the 2.5–5-km zones (RR = 10.0, 95%CI = [7], [1]–[14,2] and RR = 1.7, 95%CI = [1,4–2,0], respectively). A sensitivity analysis, which excluded land-owned parcels from these incidence calculations, had no impact on the magnitude and significance of our RR findings; it also showed that distance of contaminated versus non-contaminated parcels from the nearest QFPF (15.2 km versus 16.5 km) did not differ significantly. In addition, we observed a gradient of decreasing incidences with distance around QFPF and contaminated land parcels, but not for non-contaminated land parcels.

**Table 4:** Population, human cases and human incidence around QFPFs, contaminated land parcels and non-contaminated land parcels within three distance classes in 2009.

Distance class	0–2.5 km	2.5–5 km	5–10 km
QFPFs (n = 107)			
Population, n	602,395	2,077,560	4,195,780
Number of human cases	618	713	826
Incidence*	103	34	20
RR	5.2	1.7	*Reference*
Contaminated land parcels (n = 2464)			
Population, n	3,091,250	3,805,280	5,582,560
Number of human cases	1174	670	409
Incidence*	38	18	7
RR	5.2	2.4	*Reference*
Non-contaminated land parcels (n = 505)			
Population, n	844,760	1,533,380	3,723,060
Number of human cases	32	163	460
Incidence*	4	11	12
RR	0.3	0.9	*Reference*
RR QFPF vs contaminated land parcels (95% CI)	2.7 (2.5–3.0)	1.9 (1.8–2.2)	2.7 (2.4–3.0)
RR contaminated vs non-contaminated land parcels (95% CI)	10.0 (7.1–14.2)	1.7 (1.4–2.0)	0.6 (0.5–0.7)

*incidence = number of human cases*100000/population.

## Discussion

Our findings - a close temporal association between land application of manure and onset of illness in notified human cases, a high percentage of notified human cases geographically associated with contaminated land parcels, and a significantly higher incidence of notified human cases around contaminated versus non-contaminated land parcels – suggest that field application of manure played a significant role in the transmission of Q-fever in the Dutch Q-fever epidemic of 2006–2010.

### Temporal Sequence of Events

The incubation period of Q-fever may range from two weeks to almost 50 days, depending on the *C. burnetii-*inoculating dose, with an average incubation period of three weeks [27], [28], [29]. Peak of onset of illness in notified human cases, based on our data, lagged the peak of manure application, that on its turn lagged the peak in lambing. While manure application extended well into April, lambing took a sharp decline in April and fell to low levels. Given aforementioned average incubation period of three weeks, onset of illness in notified human cases seems more plausibly associated with application of manure than with lambing. While this does not prove a causal link, and does not diminish the key role of lambing, it may serve as a strong indicator that field application of manure played a contributing role in the transmission of Q-fever during the Dutch epidemic.

The time between the peak in lambing and the first peak in manure transport was about one month. This period is well below the limit of three months or more after which manure may be considered *C. burnetii*-free [21]. Manure applied shortly after the lambing period likely contains large amounts of viable *C. burnetii* from infected birth products that remain in the litter after lambing. Our data show that 78% of contaminated manure transports from QFPFs actually took place within one month after lambing, suggesting high concentrations of viable *C. burnetii* in land-applied manure. The bacteria are highly resistant to chemical agents and physical conditions and can survive for long periods in the environment [30]. When contaminated manure is spread over the land, the bacteria may be aerosolized with dust particles and thus transported to nearby residential locations. The second peak of manure transports, lagging the annual lambing season by several months, was not followed by a peak in human cases, which may readily be explained by much lower or absent levels of viable bacteria in the manure due to lack of (contaminated) birth products and a presumed lower degree of bacterial shedding. This notion is supported by a Dutch study that found high concentrations of *C. burnetii* DNA but no viable bacteria in manure samples taken in autumn on two QFPFs [21].

### Temporo-spatial Association between Human Cases and QFPFs or Contaminated Manure Destinations

Contaminated land parcels were associated with the highest percentages of human cases across all distance ranges and years. Percentages were lower for QFPFs, and lowest for non-contaminated land parcels (Table 1). Linking human cases only to QFPFs, and subdividing these farms into those with land and those without, the percentage of human cases associated with farms with land was always larger (Table 2). This excess percentage may be explained by the additive effect of manure spread in these farms’ vicinity, lending further evidence to our hypothesis.

Human incidence decreased with distance from QFPFs and contaminated land parcels in 2009; such decrease was not observed around non-contaminated land parcels (Table 4). The gradient observed for QFPFs and contaminated land parcels, but not for non-contaminated land parcels, is likewise suggestive of applied manure contributing to human Q-fever cases. Incidence around QFPFs was higher than around contaminated land parcels at all distance classes. Although no data are available to prove it, this might be due to lower bacterial loads in contaminated land parcels compared to QFPFs, possibly as a consequence of storage of manure over several weeks.

Human incidence around contaminated land parcels, in turn, was higher than around non-contaminated land parcels, except for the 5–10 km zone where spatial overlap with contaminated land parcels or QFPFs may have played a role. The observed incidence gradients and calculated risk ratios support our hypothesis.

Our study had several limitations. First, for lack of more precise data, we used the date of manure transport as a proxy for the date of field application. We feel sure that this is a good approximation to reality, as the use of deep litter manure as a fertilizer is mostly limited to the start of the crop growing season in spring (March–April), and – to a lesser degree – to the period from August–September when winter grain is sowed. Field application in spring thus closely follows lambing, when risk of contamination is highest, and - given the short time window between lambing and field application of manure - any effect caused by a potential difference between the time of transport and the actual time of field application should be negligible. Second, for land-owning farmers, we had no data on the exact dates of field application of manure to their own land. However, we had full access to the database of DR. Based on this database, we found that 59% of all manure transports from QFPFs took place within three months after lambing and thus met the criteria for being categorized as contaminated. Out of these, 78% (i.e. 46% of all transports) took place within an even shorter time frame of one months after lambing. Following the same argumentation regarding the seasonality of land application, we had no reason to assume that land-owing farmers significantly diverged from that time pattern. Also, the database showed there was no ‘second wave’ of transports that we would have expected to follow the peak in lambing by three months, if all transports from QFPFs had been delayed in accordance with the mandatory moratorium prohibiting transport of manure from contaminated farms during a period of 90 days following lambing. Third, our distinction of contaminated versus non-contaminated manure is based on recent literature evidence [21] and epidemiological reasoning, i.e., we had no laboratory data confirming the contamination status for land parcels included in our analyses. According to our three-month criterion, we considered manure as contaminated if, and only if, it was transported (and applied) within a period of three months after lambing. This is in accordance with the stringent government regulations from June 2008 onwards, prohibiting transport of manure from contaminated farms during a period of 90 days following lambing [2]. Thus, misclassification of contaminated manure as non-contaminated seems unlikely. An experimental Dutch study argues that survival time of *C. burnetii* in stored manure would be unlikely to exceed a very short period, possibly no more than two weeks [21]. Our three-month criterion thus may have led to systematic misclassification of non-contaminated manure as contaminated. However, based on data from that experimental study, we calculated that infectiousness of manure – under less favourable environmental and weather conditions – may well exceed that two-week period and extend to periods of up to three months. Moreover, as we explained above, 78% of all contaminated transports took place within a period of one month following lambing, even from QFPFs, in spite of aforementioned government regulations, making misclassification less likely in general. In addition, we performed a sensitivity analysis, where we used a one-month criterion as a cut-off. This had no significant impact on our results. Fourth, potential confounders, such as farm management practices and local environmental conditions, have not been included in our analyses. While we cannot entirely rule out bias from these factors, we feel they were of minor importance to our outcomes, particularly to the difference we found in the incidence of human cases surrounding contaminated versus non-contaminated land parcels. We have no reason to assume that the increased relative risk associated with contaminated land parcels can be explained by systematic error, as the choice for application of contaminated versus non-contaminated manure to any land parcel would unlikely have been influenced by any of these potential confounders. Since geographical vicinity of contaminated land parcels to QFPFs may be an exception to this assumption, we performed a sensitivity analysis where we recalculated incidences and corresponding relative risks excluding all land-owned parcels. Incidences and corresponding relative risks were similar to those in Table 4 and vicinity is considered unlikely to account for the increased relative risk associated with contaminated land parcels. In addition, we calculated the average distance of contaminated land parcels and non-contaminated land parcels to the nearest QFPF. Our data show that for all manure transports, contaminated land parcels, on average, were located at 15,164 m from the nearest QFPF (min = 17 m, max = 83,407 m, SD = 18,269 m), while non-contaminated land parcels were located at a mean distance of 16,498 m from the nearest QFPF (min = 11 m, max = 82,620 m, SD = 17,882 m).

Our study raises important issues, particularly in light of government regulations intended to curb transmission of Q-fever during the Dutch epidemic in 2006–2010. From June 2008, following that year’s lambing season and peak Q-fever transmission period, the Dutch government took measures in order to reduce the transmission of *C. burnetii* [9]. According to these regulations, QFPFs were prohibited to remove manure from the affected premises for 90 days from veterinary notification of Q-fever. In February 2009, the 90-day moratorium for manure removal following notification was adapted to a 30-day moratorium counted from the end of the lambing season, and measures were extended to include all dairy-goat farms, regardless of their Q-fever status. Besides, a hygiene protocol became mandatory, demanding coverage of manure during storage and transport and instant underplouging of manure at the moment of spreading on farming land. Yet, we observed that the vast majority of transports, regardless of Q-fever status of the farm and year under study took place within one month after lambing. Moreover, we do not observe a temporal shift in the peak of transports in 2009 compared to earlier years, suggesting that farmers were not aware of current legislation, or did not act in accordance with legislation. Whether disregard of regulations was driven by negligence, indifference or other factors is open to speculation. Given manure’s potential for zoonotic transmission, transparency regarding its handling, storage and application is indispensable for effective surveillance and communicable disease control. As to the investigation of future zoonotic outbreaks, our study argues in favour of a one-health approach, where data on people and animals would need to be collected in an integrated, multidisciplinary fashion in order to be able to answer questions about sources and underlying causes of such outbreaks. Findings from our study should be corroborated by future research, which may include reinvestigation of past clusters or outbreaks of Q-fever including suspect land parcels as potential sources, experimental studies, or sampling of aerosols and soil from and around suspect land parcels, among others. Meanwhile, findings from our study argue for a reassessment of current regulations regarding the handling, storage and application of manure with zoonotic potential. Additionally, the enforcement and control on manure treatment and manure transport, including transport to own land parcels, should be improved. To improve observance of regulations regarding the handling and processing of manure, relevant authorities should intensify their enforcement and control efforts, while farmers should receive adequate public health education as to the causes and consequences of Q fever and other zoonosis, to improve farmers’ understanding and compliance.

## Conclusion

Our findings deliver evidence that, apart from Q-fever positive farms, land-applied contaminated manure may be a significant source of human Q-fever:

- The temporal sequence of events, where lambing is followed by field application of manure, and manure application is followed by illness onset in human cases, is compatible with, and suggestive of, a contributing role of manure, given the average incubation period of human Q-fever;
- A higher percentage of human cases were temporo-spatially linked with contaminated land parcels than with Q-fever positive farms (QFPFs);
- Incidence of human Q-fever cases was significantly higher around contaminated land parcels than non-contaminated land parcels.
- Incidence of human Q-fever cases around contaminated land parcels decreased with distance, suggesting an exposure-response relationship, while incidence around non-contaminated land parcels did not;
- A higher percentage of human cases were temporo-spatially linked with QFPFs with land than QFPFs without land.
